# *PRKAR1A*-negative familial Cushing’s syndrome: two case reports

**DOI:** 10.1186/s13256-015-0757-7

**Published:** 2015-12-01

**Authors:** Lee Ling Lim, Normayah Kitan, Sharmila Sunita Paramasivam, Jeyakantha Ratnasingam, Luqman Ibrahim, Siew Pheng Chan, Alexander Tong Boon Tan, Shireene Ratna Vethakkan

**Affiliations:** 1 Division of Endocrinology, Department of Internal Medicine, University of Malaya Medical Center, Lembah Pantai, 59100 Kuala Lumpur, Malaysia; 2 Division of Endocrine Surgery, Department of Surgery, Putrajaya Hospital, Putrajaya, Malaysia

**Keywords:** ACTH-independent Cushing’s syndrome, Primary pigmented nodular adrenocortical disease (PPNAD), Carney’s complex (CNC), *PRKAR1A* gene

## Abstract

**Introduction:**

Determining the etiology of Cushing’s syndrome is very challenging to endocrinologists, with most of the difficulty arising from subtype differentiation of adrenocorticotropic hormone–dependent Cushing’s syndrome. We present the pitfalls of evaluating a rare cause of adrenocorticotropic hormone–independent Cushing’s syndrome in the transition period between adolescence and adulthood.

**Case presentation:**

A sibling pair with familial isolated primary pigmented nodular adrenocortical disease is described. The index case, a 20-year-old Chinese woman, presented with premenopausal osteoporosis with T12 compression fracture and young hypertension. Biochemical analysis confirmed adrenocorticotropic hormone–independent Cushing’s syndrome (elevated 0800 h plasma cortisol 808 nmol/L with suppressed adrenocorticotropic hormone level <5 pg/ml). Computed tomography of her adrenal glands revealed a 0.7-cm left adrenal hypodense nodule. After a left adrenalectomy, she had residual hypercortisolism (progressive weight gain, new T10 compression fracture, and not glucocorticoid-dependent postoperatively). Completion of contralateral adrenalectomy was performed upon recognition of typical histologic characteristics of primary pigmented nodular adrenocortical disease found in an initial left adrenalectomy specimen. Similarly, her younger brother developed adrenocorticotropic hormone–independent Cushing’s syndrome at age 18 years, with typical cushingoid habitus, but no osteoporosis or hypertension. His adrenal computed tomographic scans showed micronodularities over bilateral adrenal glands. He was successfully treated with bilateral adrenalectomy. Screening for Carney’s complex and *PRKAR1A* gene mutation was negative. Signs and symptoms of Cushing’s syndrome resolved after bilateral adrenalectomy for both patients. They were placed on lifelong glucocorticoid and mineralocorticoid replacement therapy and long-term surveillance for Carney’s complex.

**Conclusions:**

The cases of these two patients illustrate the difficulties involved in diagnosing primary pigmented nodular adrenocortical disease, a variant of adrenocorticotropic hormone–independent Cushing’s syndrome that is managed with bilateral adrenalectomy. A high index of suspicion for this disease is needed, especially in adolescents with adrenocorticotropic hormone–independent Cushing’s syndrome who have a significant family history, features of Carney’s complex, and no resolution of Cushing’s syndrome after unilateral adrenalectomy. Patients with primary pigmented nodular adrenocortical disease can either have bilateral/multiple adrenal nodules or normal adrenal glands visualized by computed tomography. Long-term surveillance is imperative in patients with confirmed Carney’s complex and in those who have not undergone complete genetic testing to exclude this hereditary disorder.

## Introduction

Endogenous Cushing’s syndrome (CS) is a rare disorder with an incidence of 1.2 cases/1 million/year [1]. Primary pigmented nodular adrenocortical disease (PPNAD) is a very rare cause of endogenous CS in adults, but it is more common in adolescence and early adulthood [2]. We report the cases of two siblings with CS secondary to familial isolated PPNAD and discuss pitfalls of diagnosing CS in patients in the transition zone between pediatric and adult endocrinology.

## Case presentations

### Patient 1

A 20-year-old Chinese woman presented to an orthopedic surgeon at our hospital with persistent back pain. She was then referred to an endocrinologist for assessment of vertebral fragility fracture. She complained of progressive weight gain (7 kg within 4 weeks), acne, easy bruising, proximal myopathy, and oligomenorrhea of 1 years’ duration. Her physical examination revealed that she was hypertensive (blood pressure 150/100 mmHg) and short in stature (height 1.47 m), and her body mass index (BMI) was 22.2 kg/m^2^. She had thin skin; dorsocervical/supraclavicular fat pads; central obesity; acanthosis nigricans; moon facies; and purplish striae on the abdomen, inner thighs, and popliteal fossae. She was not hirsute. Her secondary sexual characteristics and visual fields were normal. A provisional diagnosis of hypercortisolism was made.

**Fig. 1:**
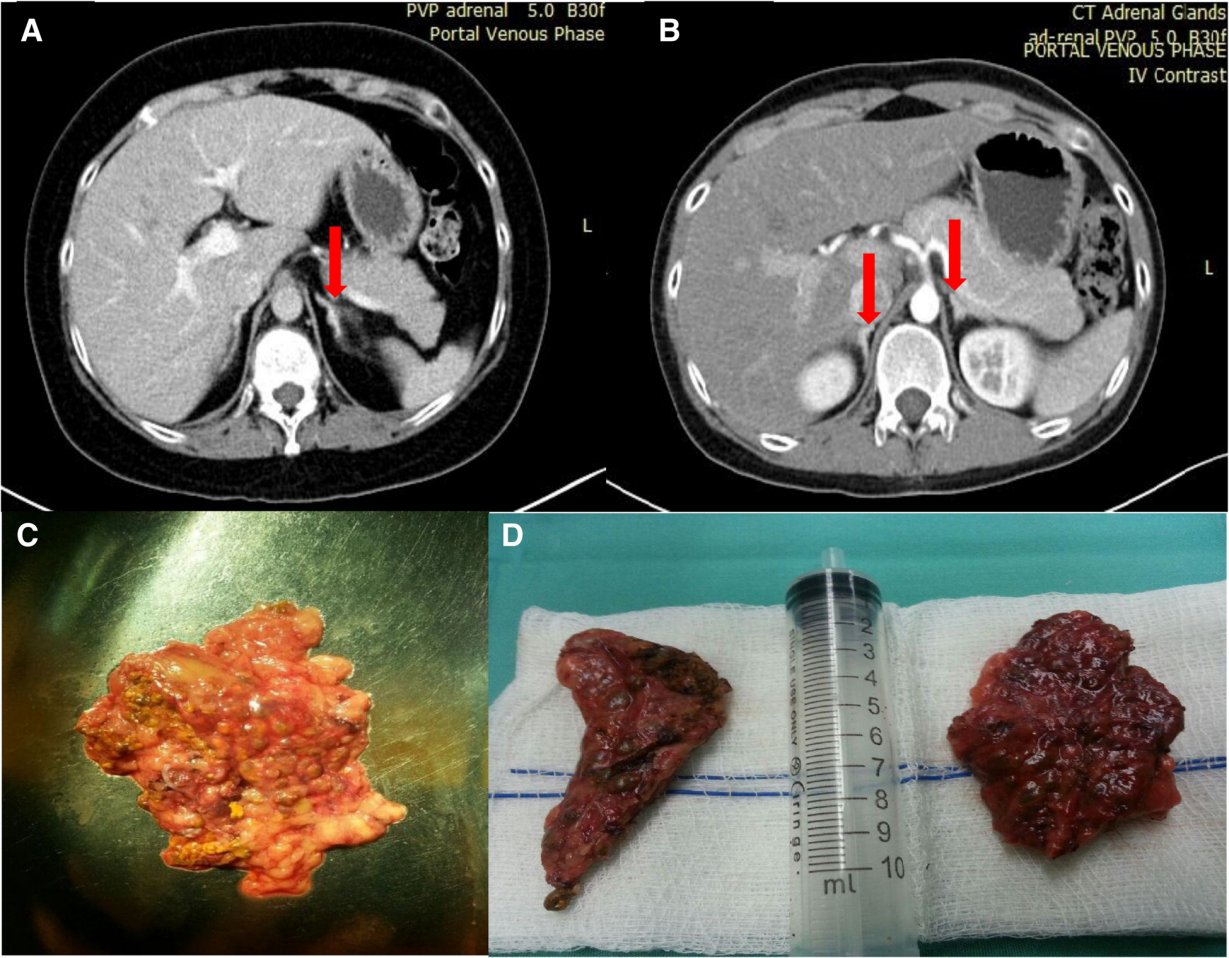
Computed tomography of the adrenal glands and gross pathology of the lesions. Patient 1: Computed tomographic scan of her adrenal glands shows a nodular left adrenal gland with hypodense lesions (*red arrow*) and a normal right adrenal gland (**a**), and histologic specimen shows cut surface of left adrenal gland with multiple brown nodules (**c**). Patient 2: Computed tomographic scan shows a hypodense micronodular appearance of both adrenal glands (*red arrows*) (**b**), and gross histologic specimens reveal adrenal hyperplasia with multiple pale brown nodules (**d**)

Her blood test results excluded diabetes mellitus and electrolyte abnormalities. Her hormone tests revealed hypercortisolemia (0800 h plasma cortisol 808 nmol/L), and a suppressed ACTH level <5 pg/ml. Her total serum testosterone was mildly elevated (Table 1). ACTH-independent CS was confirmed after a 48-h, 2-mg, low-dose dexamethasone suppression test (LDDST) failed to suppress endogenous cortisol secretion (0800 h post-LDDST plasma cortisol 621 nmol/L). A 0.7-cm left adrenal hypodense nodule was identified by performing adrenal computed tomography (CT) and was reported as an adenoma (Fig. 1a). An x-ray of her thoracolumbar spine disclosed a T12 compression fracture. Dual-energy bone densitometry (DXA) revealed low bone mineral density (BMD) with Z-scores of −4.5 (L1-L2) and −3.2 (femoral neck).

**Table 1:** Investigation results

Investigations	Patient 1	Patient 2	Reference range
Liddle’s test	N/A	24-h UFC (predexamethasone) 3923 nmol/L	58–805 nmol/L
		24-h UFC (postdexamethasone) 4168 nmol/L	
HbA1c (NGSP)	5.1 %	5.1 %	<6.5 %
fT4	12.9 pmol/L	16.7 pmol/L	11–23 pmol/L
TSH	0.67 mIU/L	0.72 mIU/L	0.5–5.5 mIU/L
IGF-1	268 ng/ml	282 ng/ml	116–350 ng/ml (patient 1)
			193–731 ng/ml (patient 2)
GH	2.3 ng/ml	0.2 ng/ml	0.0–3.0 ng/ml
LH	1.4 IU/L	5.8 IU/L	Follicular phase 1.9–12.5 IU/L
			Midcycle 8.7–76.3 IU/L
			Luteal phase 0.5–16.9 IU/L
			Male 1.5–9.3 IU/L
FSH	2.2 IU/L	8.8 IU/L	Follicular phase 2.5–10.2 IU/L
			Midcycle 3.4–33.4 IU/L
			Luteal 1.5–9.1 IU/L
			Male 1.4–18.1 IU/L
Testosterone	3.4 nmol/L	13.0 nmol/L	Female 0.5–2.6 nmol/L
			Male 9.3–23.0 nmol/L
DHEA-S	0.07 μg/dl	0.04 μg/dl	Female 0.02–0.30 μg/dl
			Male 0.10–0.40 μg/dl
Estradiol	153 pg/ml	N/A	Follicular phase 20–144 pg/ml
			Midcycle 64–357 pg/ml
			Luteal phase 56–214 pg/ml
Prolactin	123 mIU/L	127 mIU/L	Female 59–619 mIU/L
			Male 44–373 mIU/L
Thyroid US	Normal	Well-defined right thyroid cystic nodule with soft tissue component (1.1 × 1.0 × 1.4 cm), increased peripheral vascularity	

*N/A* not applicable *HbA1c* hemoglobin A1c, *NGSP* National Glycohemoglobin Standardization Program, *DHEA-S* dehydroepiandrosterone sulfate, *US* ultrasound, *IGF-1* insulin-like growth factor 1, *GH* growth hormone, *LH* luteinizing hormone, *FSH* follicle-stimulating hormone, *fT4* free thyroxine, *TSH* thyroid-stimulating hormone, *UFC* urinary free cortisol

**Fig. 2:**
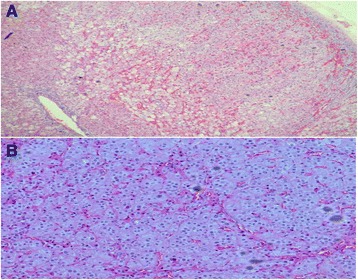
Adrenal histology [hematoxylin and eosin stain; original magnifications ×4 (**a**) and ×10 (**b**)]. Multiple nodules (2–5 mm in diameter) containing brown pigment can be seen in the adrenal cortex, composed of cells with round to ovoid nuclei with eosinophilic cytoplasm. Many cells with vacuolated, foamy cytoplasm were noted, also in addition to marked vascular congestion. Immunohistochemistry showed positivity for synaptophysin and vimentin, focal positivity for chromogranin A and S100, and a negative result for cytokeratin antibody MNF116

Preoperatively, she was treated with spironolactone (for hypertension and edema), ketoconazole (to inhibit steroidogenesis), and teriparatide, cholecalciferol, and calcium supplements (for low BMD and vertebral fracture). A left retroperitoneoscopic adrenalectomy was performed 4 weeks later. Her hypertension and edema resolved after surgery. She was commenced on physiologic hydrocortisone replacement therapy in anticipation of transient hypocortisolism due to suppression of the contralateral adrenal gland. One month postoperatively, her 0800 h serum cortisol measured before the morning dose of hydrocortisone was 384 nmol/L. She continued to gain weight, had persistent oligomenorrhea, and developed a new T10 compression fracture with 2-cm height reduction. Reevaluation after cessation of hydrocortisone showed failed suppression of endogenous plasma cortisol with a 1-mg overnight LDDST (0800 h plasma cortisol 248 nmol/L). By then, histology of the excised left adrenal gland unexpectedly revealed pigmented nodular adrenocortical hyperplasia (Fig. 2). The patient’s preoperative CT was reviewed, confirming micronodularity with multiple nodules of the left adrenal gland, which had been missed initially. She had no dermatologic features (lentigines/blue nevi) or murmurs suggestive of cardiac myxoma characteristic of Carney’s complex (CNC). The results of ultrasound (US) of her breasts and pelvis, as well as of transthoracic echocardiography (TTE) and other tests performed to screen for features of CNC, were negative (Table 1).

A complete right retroperitoneoscopic adrenalectomy was scheduled. Histology confirmed the initial diagnosis of PPNAD. Lifelong hydrocortisone and fludrocortisone replacement therapy was commenced after surgery. Her postoperative 0800 h serum cortisol level was <6 nmol/L. Repeat DXA 2 months after surgery showed improvement of her BMD with Z-scores of −3.1 (L1-L2) and −2.6 (femoral neck). Teriparatide and cholecalciferol/calcium carbonate were continued for 18 months. She is now normotensive, has reestablished a normal menstrual cycle, and no longer has a cushingoid habitus.

### Patient 2

The younger sibling of patient 1, an 18-year-old Chinese man, presented to us 3 months after his sister’s first surgery. He had a 2-year history of purple striae and complained of rapid weight gain (4 kg in 4 weeks). His physical examination revealed that his height was 1.63 m and his BMI was 23.1 kg/m^2^. He had no lentigines/blue nevi. He was normotensive but had moon facies and purple striae (bilateral axillae, popliteal fossae, and waist). He did not have a goiter, proximal myopathy, or and dorsocervical/supraclavicular fat pads. His secondary sexual characteristics were normal, with no testicular mass evident.

His blood tests excluded diabetes mellitus and electrolyte abnormalities. His hormone tests revealed suppressed ACTH (<5 pg/ml) and failed suppression of endogenous plasma cortisol after 1-mg overnight LDDST (0800 h serum cortisol 624 nmol/L). Subsequent LDDST confirmed CS (0800 h plasma cortisol 513 nmol/L). Liddle’s test revealed a paradoxical increase in urinary free cortisol (UFC) (Table 1). DXA disclosed secondary osteoporosis [Z-scores of −3.2 (spine) and −2.5 (femoral neck)]. Adrenal CT revealed a diffusely enlarged left adrenal gland and normal-sized nodular right adrenal gland (Fig. 1b). The results of TTE and US scans of the testes as well as other screening (Table 1) for features of CNC were unremarkable. His thyroid US revealed a well-defined nodule, which fine-needle aspiration confirmed as a benign follicular nodule.

A bilateral retroperitoneoscopic adrenalectomy was performed, with ketoconazole used as bridging therapy. The results of patient 2’s histologic examination were similar to those of patient 1, establishing the diagnosis of PPNAD (Fig. 2). His postoperative 0800 h serum cortisol level was <6 nmol/L, and he was commenced on lifelong hydrocortisone and fludrocortisone replacement therapy. His signs and symptoms of CS resolved after 6 months.

### Follow-up of both patients

**Fig. 3:**
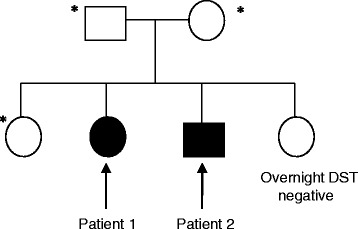
Family tree for this sibling pair. *refused screening for CS

Both patients were given a steroid card and counseled on sick-day rules. Their parents were not in a consanguineous marriage. The family tree was shown in Fig. 3. There was no family history of CNC. Genetic tests for the *PRKAR1A* mutation were sent to the National Institutes of Health with the patients’ written informed consent. The results of DNA sequencing for *PRKAR1A* gene point mutation and deletion/duplication were negative for both siblings. Unfortunately, they declined further genetic testing despite genetic counseling.

## Discussion

**Fig. 4:**
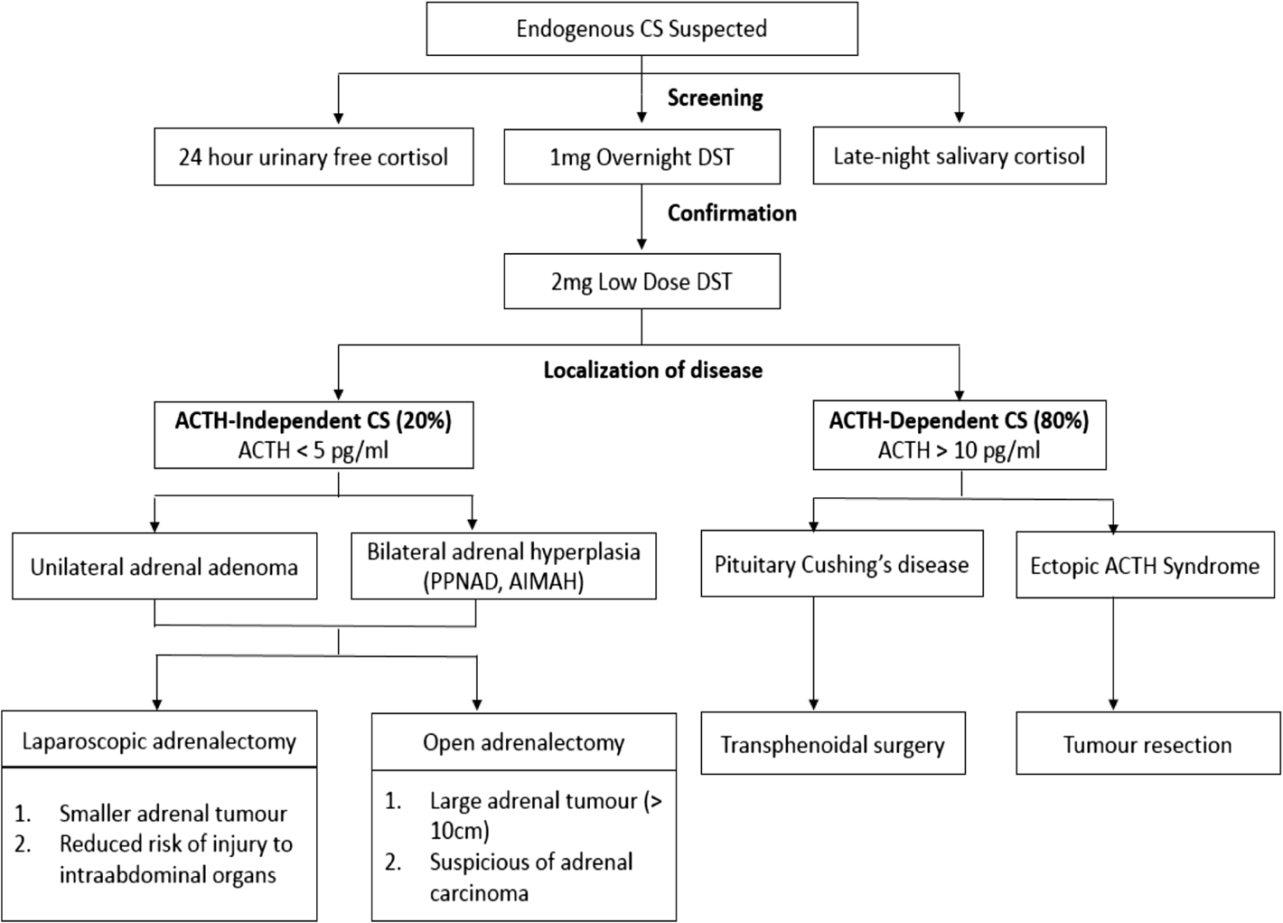
An algorithm for the diagnosis and management of Cushing’s syndrome. Surgical therapy is the mainstay of treatment in Cushing’s syndrome. Adapted from [3, 4, 19]

Determining the etiology of endogenous CS is one of the most complex challenges that confronts an endocrinologist (Fig. 4) [3, 4]. The causes of CS are broadly subclassified into ACTH-independent (20 %) and ACTH-dependent (80 %) categories. ACTH-independent CS is most commonly due to unilateral adrenocortical tumors. Only 10–15 % of ACTH-independent CS is caused by bilateral adrenal hyperplasia (BAH) [2], which comprises PPNAD and ACTH-independent macronodular adrenal hyperplasia (AIMAH). Of the two, PPNAD is more common [2]. In a 10-year adult population-based study in Denmark, only 2 (1.4 %) of 139 patients with CS were diagnosed with isolated PPNAD [1]. In children, BAH may account for a greater proportion of ACTH-independent CS. The authors of a retrospective review of 105 patients with CS (3–18 years old) found that 23.8 % had primary adrenal disease, and 84 % of these had BAH [5]. In this report, we present a rare cause of ACTH-independent CS in the transition zone from pediatric to adult endocrinology.

PPNAD, which has an autosomal dominant inheritance pattern, can be sporadic or familial (isolated or more commonly associated with CNC) [6]. It has a bimodal age distribution with peaks before age 5 years and, more commonly, in the second and third decades of life. Our two patients presented at ages 20 and 18 years, respectively.

The signs and symptoms of PPNAD are insidious yet progressive, and diagnosis is delayed in most patients [6]. Three different phenotypes of PPNAD [overt (84 %), subclinical (6 %), and latent (10 %)] have been described [6, 7]. Our sibling pair presented with overt PPNAD. Compared with other causes of CS, osteoporosis (39 % of PPNAD) and growth retardation (29 % of PPNAD) [6] are more prevalent. CS is one of the common causes of young hypertension, which is generally defined as BP ≥140/90 mmHg in patients <40 years old [8]. However, hypertension tends to occur in only 55–85 % of patients with CS [9]. Thus, in our two patients, the younger brother did not develop hypertension as seen in the older sister.

The 6-day Liddle’s test helps to differentiate PPNAD from other causes of ACTH-independent CS. Most patients have a >50 % paradoxical increase in UFC and 17-hydroxysteroid secretion due to overexpression of glucocorticoid (GC) receptors in PPNAD nodules [7, 10]. An atypical 6 % paradoxical increase in UFC was observed in patient 2, which was suggestive of PPNAD in the context of a positive family history.

In the context of ACTH-independent CS, PPNAD should be suspected in patients with multiple nodules in one or both adrenal glands, bilateral solitary nodules on either side [11], unilateral/bilateral enlargement of the adrenal gland, or normal adrenal glands bilaterally visualized by imaging [12]. About half of PPNAD patients have normal-sized adrenal glands seen on CT scans, as in our sibling pair [12, 13]. Other differential diagnoses for bilateral adrenal masses/abnormalities in the setting of suppressed ACTH are AIMAH (usually associated with a macronodular appearance), bilateral cortisol-secreting adenomas/adenolipomas [10], or a unilateral adrenal adenoma with contralateral nonfunctioning incidentaloma. Multiple micronodules observed on CT scans give rise to a “strings of beads” appearance, which is typically seen between ages 12 and 18 years [12]. After age 18 years, patients with PPNAD can even have adrenal macronodules (2–3 cm), which are indistinguishable radiologically from AIMAH and pituitary Cushing’s syndrome with BAH [12].

Adrenal CT and/or magnetic resonance imaging do not afford the ability to determine the functionality of adrenal lesions in ACTH-independent CS with bilateral adrenal masses. Adrenal venous sampling (AVS) may serve as an adjunct in this setting, helping in the decision-making of whether a unilateral or bilateral adrenalectomy is required. Young *et al.* performed AVS in ten patients and reported that an adrenal vein to peripheral vein cortisol gradient >6.5 was consistent with a cortisol-secreting adenoma and that a cortisol lateralization ratio of ≤2.0 was suggestive of bilateral cortisol hypersecretion [14]. AVS therefore has a potential role in the management of ACTH-independent CS, especially when CT shows bilateral adrenal nodularity. However, limited experience (due to the rarity of this condition), variations in protocol, and interpretation of results (successful catheterization and lateralization) currently hamper the routine use of AVS in ACTH-independent CS.

The characteristic macroscopic appearance of PPNAD is small to normal-sized adrenal glands with multiple small cortical pigmented nodules. Microscopically, the nodules consist of large cortical cells with eosinophilic cytoplasm, large hyperchromatic nuclei and prominent nucleoli, and atrophy between nodules [15]. Synaptophysin immunostaining helps to distinguish PPNAD nodules from surrounding normal adrenal cortex. Our patients’ histologies were typical of PPNAD.

Given its rarity, PPNAD might be overlooked. It is often recognized postoperatively based on histology. In retrospect, there were a few salient points to suggest bilateral adrenal disease rather than a unilateral adrenal adenoma in patient 1. Review of her initial adrenal CT showed micronodularities, especially over the left adrenal gland, which is not seen in patients with unilateral adrenal adenoma. Also, despite partial resolution of her symptoms, she had residual hypercortisolism; that is, she continued to gain weight, developed a new T10 compression fracture, and never had GC deficiency postoperatively. Our experience has taught us that all efforts should be made to look for other features of CNC and to take a detailed family history, focusing on CNC in young patients with ACTH-independent CS.

CNC is a multiple neoplasia syndrome associated with clustering of skin tumors and pigmented lesions, myxomas, schwannomas, and endocrine tumors. PPNAD is the most common endocrine tumor associated with CNC, occurring in 60 % of patients with CNC [16]. Recognition of CNC is crucial for genetic screening recommendation, as it is an autosomal dominant trait disorder [17]. In addition, lifelong screening for associated features is indicated in patients diagnosed with CNC [17].

A definite diagnosis of CNC is made if at least two major criteria are present or one major criterion is met in an affected first-degree relative with CNC, or if the index case or relative has a *PRKAR1A* mutation [15]. The major criteria for a diagnosis of CNC are listed below [17]:

- Spotty skin pigmentation over the lips, conjunctiva, inner and/or outer canthi, and vaginal or penile mucosa
- Cutaneous, mucosal myxoma
- Cardiac myxoma
- Breast myxomatosis
- Endocrine overactivity (for example, PPNAD, acromegaly)
- Large-cell calcifying Sertoli cell tumor (LCCSCT)
- Thyroid carcinoma or multiple hypoechoic nodules on thyroid US
- Psammomatous melanotic schwannoma
- Multiple blue nevi
- Multiple breast ductal adenoma
- Osteochondromyxoma

The *PRKAR1A* gene, located on chromosome 17q22-24, encodes the regulatory type Iα subunit of protein kinase A, an inactivating mutation that is responsible for the disease in nearly half of CNC kindred [16]. Phosphodiesterase 11A (*PDE11A* gene) and phosphodiesterase 8B (*PDE8B* gene) mutations, as well as chromosome 2p16 mutations, have also been identified in patients with isolated PPNAD [18].

The results for DNA sequencing for *PRKAR1A* gene point mutation and deletion and/or duplication were negative in our sibling pair. Unfortunately, the patients’ family declined Whole Exome Sequencing (WES), the next step in detailed DNA analysis of all chromosomes. Among patients with CNC, 30 % do not have a *PRKAR1A* gene mutation. Salpea *et al.* demonstrated *PRKAR1A* haploinsufficiency secondary to 17q24.2-q24.3 deletions surrounding the affected gene in 21.6 % of *PRKAR1A* mutation-negative patients [18].

In the absence of a *PRKAR1A* gene mutation, our patients do not fit the criteria for CNC. At present, they each meet only one major criterion. The most likely diagnosis is familial isolated PPNAD. As they declined WES, it is still possible that they have CNC but have not yet developed associated features enumerated in Stratakis *et al.*’s major criteria [17].

The treatment of choice for PPNAD is bilateral adrenalectomy, especially in overt PPNAD as in our sibling pair. Laparoscopic adrenalectomy is favored over an open surgical method in this era, as it is associated with shorter hospital stay, fewer perioperative complications, and earlier return to normal activity [19]. In Carney and Young’s series of 88 patients, 65 % were cured after bilateral adrenalectomy [7]. Of the patients who underwent subtotal and/or unilateral adrenalectomy, 35 % had persistence and/or recurrence of CS and required completion of total adrenalectomy [7]. However, the aforementioned retrospective review found that subtotal and/or unilateral adrenalectomy might be considered in asymptomatic or mildly symptomatic patients with PPNAD.

As we were unable to exclude non-*PRKAR1A* gene mutations in our patients, we determined that future management should incorporate long-term surveillance for CNC. The presence of a benign follicular nodule in patient 2 warrants periodic thyroid US at 6–18-month intervals [20]. If thyroid carcinoma develops, it tends to present at a younger age and with a higher incidence of multifocality, invasion, and local recurrence [21]. Regular US of the testes is imperative for patient 2, as males with CNC tend to develop LCCSCT, which blocks seminiferous tubules, causing infertility. Serial DXA will be performed to monitor the expected improvement in BMD in both patients postoperatively. In patient 1, there was only partial improvement of her BMD after surgery. We hope that 18-month therapy with teriparatide will help rebuild bone density and eventually result in a normal peak bone mass. Bisphosphonates were not used in her case, owing to concern with regard to its safety in young women of childbearing age. Monitoring for Nelson’s syndrome is crucial for patients who undergo bilateral adrenalectomy. Of note, Carney and Young did not report any case of Nelson’s syndrome after bilateral adrenalectomy in patients with CNC [7].

## Conclusions

The two reported cases illustrate the difficulties involved in diagnosing PPNAD, a variant of ACTH-independent CS that more commonly presents with osteoporosis in young patients and is ideally managed by bilateral adrenalectomy. A high index of suspicion is needed, especially in adolescents with ACTH-independent CS, a family history of CS, multiple and/or bilateral adrenal nodules, features of CNC, and failure of resolution of CS after unilateral adrenalectomy. The diagnosis of PPNAD in patients without a family history or an index case in a yet-to-be-identified kindred is harder. Long-term surveillance for CNC is imperative in patients with confirmed CNC and those who have not undergone complete genetic testing to exclude CNC.

## Consent

Written informed consent was obtained from the patients for publication of this case report and any accompanying images. A copy of the written consent is available for review by the Editor-in-Chief of this journal.
